# Flexibility during the COVID-19 Pandemic Response: Healthcare Facility Assessment Tools for Resilient Evaluation

**DOI:** 10.3390/ijerph182111478

**Published:** 2021-10-31

**Authors:** Andrea Brambilla, Tian-zhi Sun, Waleed Elshazly, Ahmed Ghazy, Paul Barach, Göran Lindahl, Stefano Capolongo

**Affiliations:** 1 Design and Health Lab, Department of Architecture, Built Environment and Construction Engineering (DABC), Politecnico di Milano, 20133 Milan, Italy; tianzhi.sun@polimi.it (T.-z.S.); Paul.Barach@jefferson.edu (P.B.); goran.lindahl@chalmers.se (G.L.); stefano.capolongo@polimi.it (S.C.); 2 Center for Healthcare Architecture (CVA), Division of Building Design, Department Architecture and Civil Engineering (ACE), Chalmers University of Technology, SE-412 96 Goteborg, Sweden; 3 School of Architecture and Urban Planning (AUIC), Politecnico di Milano, 20133 Milan, Italy; waleedashraf92@hotmail.com (W.E.); ahmed.ghazy@mail.polimi.it (A.G.); 4 Jefferson College of Population Health, Thomas Jefferson University, Philadelphia, PA 19107, USA; 5 School of Medicine and Law, Sigmund Freud University, 1020 Vienna, Austria

**Keywords:** flexibility, healthcare facilities, hospitals, assessment tool, COVID-19, evaluation

## Abstract

Healthcare facilities are facing huge challenges due to the outbreak of COVID-19. Around the world, national healthcare contingency plans have struggled to cope with the population health impact of COVID-19, with healthcare facilities and critical care systems buckling under the extraordinary pressures. COVID-19 has starkly highlighted the lack of reliable operational tools for assessing the level sof flexibility of a hospital building to support strategic and agile decision making. The aim of this study was to modify, improve and test an existing assessment tool for evaluating hospital facilities flexibility and resilience. We followed a five-step process for collecting data by (i) doing a literature review about flexibility principles and strategies, (ii) reviewing healthcare design guidelines, (iii) examining international healthcare facilities case studies, (iv) conducting a critical review and optimization of the existing tool, and (v) assessing the usability of the evaluation tool. The new version of the OFAT framework (Optimized Flexibility Assessment Tool) is composed of nine evaluation parameters and subdivided into measurable variables with scores ranging from 0 to 10. The pilot testing of case studies enabled the assessment and verification the OFAT validity and reliability in support of decision makers in addressing flexibility of hospital design and/or operations. Healthcare buildings need to be designed and built based on principles of flexibility to accommodate current healthcare operations, adapting to time-sensitive physical transformations and responding to contemporary and future public health emergencies.

## 1. Introduction

### 1.1. The Challenge of Hospital Flexibility in COVID-19 Pandemic and Beyond

Healthcare systems and their hospital facilities are facing huge challenges since the outbreak of COVID-19, in regards to the management of healthcare settings and building layouts [1,2], environmental contamination risks [3] and infection prevention and control operational demands [4,5]. Hospitals have been running out of space and resources to treat COVID-19 patients, whilst simultaneously caring for patients presenting with mild symptoms or who are asymptomatic, who pose an infectious risk to healthcare workers and other patients. The acceleration and stress caused by the pandemic have made the existing structural, organizational and technological challenges of worn-out and obsolete healthcare facilities even more compelling and increased the sense of urgency to redesign present facilities [6]. Healthcare facilities need to be designed in a dynamic way that can support the quadruple aim of healthcare, (4 interdependent goals consist of (1) enhancing patient experience and safety, (2) improving population health, (3) reducing costs and preventing loss of revenue, and (4) improving wellness and satisfaction of health care workers) while accommodating changes and recognizing the essential dependencies across networks of care [7]. A whole life-cycle approach to the healthcare facility operation is needed considering the rapid and constant alterations of healthcare environments resulting from transformations in medicine, technology and organizational changes [8]. Healthcare buildings must be planned and designed based on sound human factors principles and capable to accommodate current problems and needs, adapt to speedy transformations and respond to contemporary and future necessities, especially while facing emergency issues such as COVID-19 [9,10,11]. Modular and fast construction, repurposing of spaces and equipment of temporary settings have emerged as approaches to manage the urgent need for flexible and resilient solutions [12,13,14]. Contemporary healthcare infrastructures are being designed and constructed with a relatively long life span, which does not accommodate for recurrent service demand changes [15].

Flexibility in architectural design can be defined as the ability of a building to adapt to changed spatial requirements and functional solutions according to short, medium or long-time perspectives [16,17]. Flexibility represents a fundamental aspect to consider in the hospital design process, from the overall building structure planning to the functional and spatial design of care units to ensure effective emergency management [6,16] and adaptability [18]. Recent research on the topic points to key concepts such as the Open Building—the capacity of a facility to host several different functions in time [19]—or a future-proofing approach to design [20]. Flexibility is the key requirement of healthcare facilities of the future and consequently designers need to consider the unknown needs due to technological, societal and epidemiological changes [21,22].

### 1.2. Research Gap and Aims

The coronavirus crisis revealed that many modern hospitals and healthcare organizations lack the flexibility to accommodate sudden surges of patients due to unexpected healthcare situations. There is a lack of operative tools for assessing the levels of flexibility and resiliency of hospital buildings [23]. It is necessary to develop reliable and structured models and assessment frameworks to support healthcare facility managers and planners in facing disruptive challenges that require rapid modifications such as in response to COVID-19.

The aim of this research was to modify, improve and test an assessment tool that provides guidance for hospital designers to improve their proposed designs, and to be applied to existing hospitals offering deeper understanding of how the facilities satisfy the criteria and concepts of flexibility.

## 2. Materials and Methods

### 2.1. Research Design

**Figure 1:**
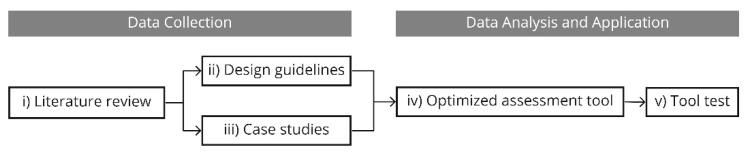
Flowchart profile of the research methodology.

The research plan followed a five-step process by collecting data through: (i) detailed literature review about flexibility principles and strategies, (ii) review of healthcare design guidelines, (iii) evaluation of international healthcare facilities case studies, (iv) critical review and optimization of an assessment tool for healthcare flexibility, and (v) usability testing method to check and compare the new with the original assessment tools (see Figure 1).

### 2.2. Data Collection through Scoping Literature Review

Scoping reviews are a traceable method of ‘mapping’ areas of research and highlighting gaps in the literature for future research. Scoping reviews are a useful tool in the ever-increasing arsenal of evidence synthesis approaches and require rigorous and transparent methods to ensure that the results are trustworthy and reproducible [24,25]. This approach summarizes the evidence available on a topic in order to convey the breadth and depth of that topic by mapping the existing literature in a field of interest in terms of the volume, nature and characteristics of the primary research and identify gaps in the existing literature [26]. In line with the methodology of scoping reviews, a formal evaluation of the quality of the studies was not undertaken. A detailed review protocol can be obtained from the primary author on request.

#### 2.2.1. Objectives of Literature Review

The objective of the scoping review was to map key concepts as a basis for a deeper understanding of the effects of facility flexibility on hospital readiness and performance while identifying gaps in our current knowledge to inform design of future hospitals [27].

#### 2.2.2. Data Sources and Search Strategy

**Figure 2:**
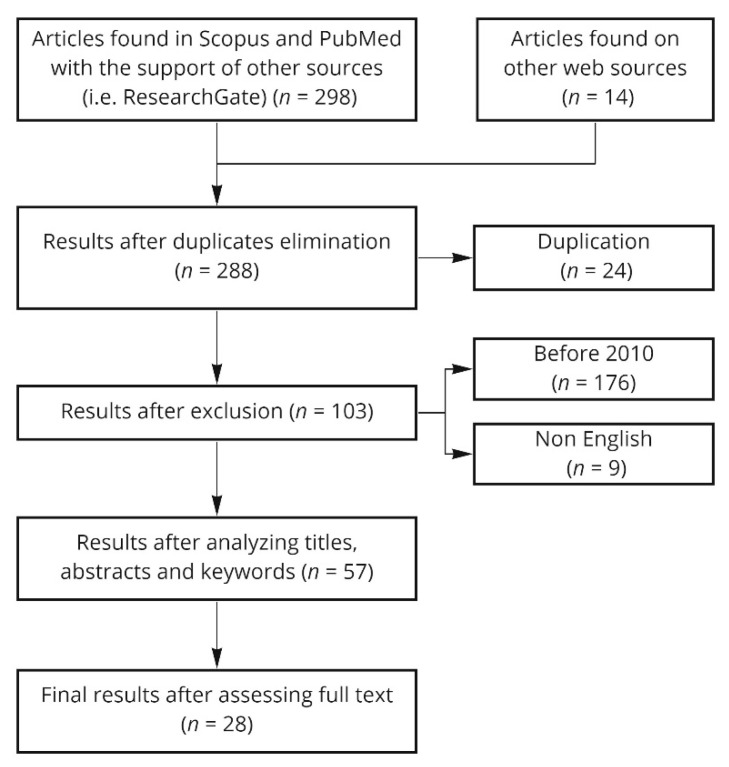
Flow diagram of literature search.

The initial search was undertaken on studies published between January 2000 and June 2021. The review of the literature was conducted by identification of search string keywords and appropriate Boolean operators (i.e., OR, AND) in databases Scopus, PubMed (including Medline), Researchgate with supplementary searches on Google Scholar. The databases were selected to be comprehensive and to cover a broad range of disciplines. Definitions and principles of flexibility were collected. In the stage of literature review (see Figure 2), the keyword searches were conducted to identify potentially relevant studies published in English along with criteria used on screening for inclusion and exclusion (see Table 1).

The study’s initial selection for inclusion was based on the title and abstract of the studies that were reviewed to preclude waste of resources in procuring articles that did not meet the minimum inclusion criteria. Two of the authors (W.E. and A.G.) reviewed titles, references and abstracts generated by the original search against the agreed inclusion and exclusion criteria. In case of disagreement, a third researcher (A.B.) revised the items. When the title and abstract provided insufficient information to determine the relevance, a full-text copy of the article was retrieved and reviewed. For the final selection, a full-text copy of each study was examined to determine if it fulfilled the inclusion criteria.

### 2.3. Data Collection through Healthcare Design Guidelines Review

A different combination of keywords (Table 2) was used to review several international guidelines to produce a comparative framework of the most fundamental aspects to be considered while designing and planning healthcare facilities. The search focused on adaptability, convertibility, and agility to cope with emerging issues of healthcare facility design. Five English language guidelines were initially selected including:

- U.K. (DH Health Building Notes);
- Australia (Australian Healthcare Facility Guidelines);
- Canada (Canadian Healthcare Facilities);
- International guidelines (International Health Facility Guidelines authored by Total Alliance Health Partners International (TAHPI);
- Facilities Guidelines Institute Design Guidelines (FGI).

One of the limitations encountered in the process was the unavailability of a free version of the design guidelines of the U.S. (FGI), hence it was excluded and only four guidelines were examined.

### 2.4. Data Collection through Case Study Analysis

A third set of keywords was selected to identify practical examples of hospital facilities to be analyzed. We choose case studies with a promising level of future-proofing that were recently completed or under construction. The case studies were chosen across varied geographies and different scales and evaluated with a critical lens focusing on flexibility and resiliency (Table 3).

Seven case studies (four European projects, one Asian project, one North American project and one Latin American project) of recent healthcare facilities were selected and analyzed with regard to flexibility and future-proofing approach, the architectural design and spatial organization/layout as well as general information such as the location, client, year of start and completion, collaborators, area and budget to establish a detailed overview for each one of the case studies.

The seven selected case studies were:

- Case study 1 (CS1): Hospital Südspidol, Esch sur Alzette, Luxembourg;
- Case study 2 (CS2): Massachusetts General Hospital (Lunder Building), Boston, MA, USA;
- Case study 3 (CS3): Machala Fluid Hospital, Machala, Ecuador;
- Case study 4 (CS4): The Sammy Ofer Heart Building, Tel Aviv, Israel;
- Case study 5 (CS5): New Karolinska Hospital, Stockholm, Sweden;
- Case study 6 (CS6): Aarhus University Hospital, Aarhus, Denmark;
- Case study 7 (CS7): New Martini Hospital, Groningen, Netherlands.

We used the Open Building Assessment Tool (OBAT), a widely used evaluation tool to evaluate each case study in terms of eight evaluation parameters: shape, structure, facade, building plant, expandability, restrictions and technologies with a grading system that allows for each parameter a score between 0 and 10 points [19,28]. The tool offers insights into the level of flexibility of each case study through their rankings on the OBAT and the ability to extract new principles not mentioned previously in the theoretical sections.

### 2.5. Tool Modification and Review

The modified assessment tool was developed through a critical review of the OBAT framework to highlight the strengths and weaknesses of each parameter of the original version. The review was conducted at the evaluation parameter level as well as on the measurement variable levels. Our analysis resulted in a modified version of the tool—Optimized Flexibility Assessment Tool (OFAT).

### 2.6. Tool Usability Testing on Case Studies

The OFAT framework was tested on two case studies of selected healthcare facilities to validate the updated instrument. The aim was to check the usability and simultaneously compare the scores of each evaluation parameter on both the original tool and the modified tool versions.

## 3. Results

### 3.1. Search Results

The initial literature search identified several articles for full-text review after meeting the eligibility and inclusion criteria and underwent a full-text abstraction. Because of the heterogeneity of the study designs, participants and outcome measures, a meta-analysis was not feasible. The full list of articles from identification to final inclusion is represented by 28 papers reported in Table S1.

The search focused on the following issues: *flexibility of space in healthcare facilities, strategies of flexibility and its impact on hospitals*. Flexibility was the most emerging and trending principle in healthcare facilities including a detailed explanation of the levels and types. In addition, we extracted from the literature the typological-spatial strategy from the different levels and types of projects.

#### 3.1.1. Definitions of Flexibility

Pati et al. found that flexibility in healthcare design depends on the perspectives patients, managers and administrators, and professionals [29]. Patients perceive flexibility regarding improved personalized care, while nursing staff perceive it mainly in operational terms. Managers and administrators perceive flexibility regarding staff management, patient care management and resource provision etc. Professionals such as architects and engineers perceive flexibility in terms of the space functionality and its proximity to other spaces, patient well-being and comfort, light, ventilation and structural grids etc.

Pati and colleagues define the three aspects of flexibility are adaptability, convertibility, and expandability [29]. A similar classification is used by Agre and Landstad, and Bjørberg and Verweij [30,31]. “Adaptability or flexibility to adapt” is the ability of the hospital infrastructure to accommodate changing requirements of healthcare without changing the environment. “Convertibility or flexibility to convert” is the ability of the healthcare infrastructure to convert according to the changing requirements of the facility with minor changes to the existing structure at a reasonable cost. “Expandability or flexibility to expand” is the ability of the hospital infrastructure to grow vertically or horizontally according to the shifting requirements of healthcare. Flexibility must be considered both from the architectural and from facility management points of view [32,33].

#### 3.1.2. Impact of Space Flexibility

Patient safety and staff efficiency are two of the essential factors for integrating space standardization and flexibility [34]. Pati et al. identified space flexibility aids in securing the future of the facility by guaranteeing a flexible environment that adapts to future transformation for staff to work [29]. Additionally, Ahmad and colleagues highlighted the impact of flexibility on staff and patients. This can happen both in terms of space flexibility, which can save time [35], reduce errors [36], reduce stress [29], reduce traveling distances [29,37,38,39], and so on, and space standardization, which can reduce errors [38], adapt needs [40], improve care [38,41], easily reuse facility [42], reduce space required [39], and so on. Standardization helps in reducing costs, easing mental workload, making errors and deviations from work easier to detect; standardization also enables the exchange of skills between different organizations, consequently enhancing staff performance.

#### 3.1.3. Levels and Types of Flexibility

Previous studies have noted that “through a better analysis of the hospital facility it is possible to identify four levels of flexibility depending on the scale of the building (i.e., hospital complex, building, functional unit or individual room). For each scale it is also possible to highlight possible types of flexibility (space or operational) achievable solely through specific typological-spatial strategies” [21]. Additionally, these levels must be subdivided by types of flexibility into constant surface spatial flexibility, variable surface spatial flexibility, and operational flexibility [21,43,44]. Facility types/(building, functional unit, rooms, etc.) require application of all types of flexibility as listed in Table 4.

#### 3.1.4. Flexibility Analysis Matrix

We developed an analysis matrix to determine which strategies are used most in a healthcare facility and to highlight which levels and types of flexibility. The matrix highlights the most common requirements for converting healthcare spaces into four levels of flexibility: from the territorial scale to an individual room.

The matrix is designed according to four levels of flexibility based on different scales as follows: hospital complex, building entity, functional unit, and individual room (see Table 5). For each level different types of flexibility are identified as follows: constant surface, variable surface, and operational flexibility, which identify the possible typological-spatial and management strategies that can be applied and achieved to assure and support future development of the healthcare facility. For example at the room level the usage of single or multiple patient rooms is widely discussed [46].

### 3.2. Flexibility Principles Matrix from the Design Guidelines Analysis

We developed the “Flexibility principles matrix for healthcare guidelines” for an overall comparison (see Table 6) and extracted 29 principles that highlight the most and the least addressed flexibility considerations in the four healthcare design guidelines.

The comparative matrix highlights the most common flexibility considerations, which are planning models, adaptability, expandability, standardization, modular design, room utilization, accessibility, and overflow design, as they are treated in some detail in the four healthcare guidelines. However, structural loading capacity, construction flexibility, equipment flexibility, interstitial floor, ceiling height, and facade design are scarcely addressed in the healthcare guidelines as they are each present in only one design guideline.

### 3.3. Flexibility Applied in Practical Healthcare Design Best Practices

Table 7 reflects the results of the seven case studies as follows:

- The assessment for Open Building flexibility total scores;
- The extent one of the evaluation parameters is fulfilled, and the points deducted due to lacking information; and,
- The range/category (out of five) of the healthcare facility, indicating whether or not they satisfy the requirements to be considered an Open Building.

None of the case studies in our analysis and evaluation scored higher than 80%, except for one healthcare facility (CS4) out of the seven case studies that was considered an ideal Open Building.

**Figure 3:**
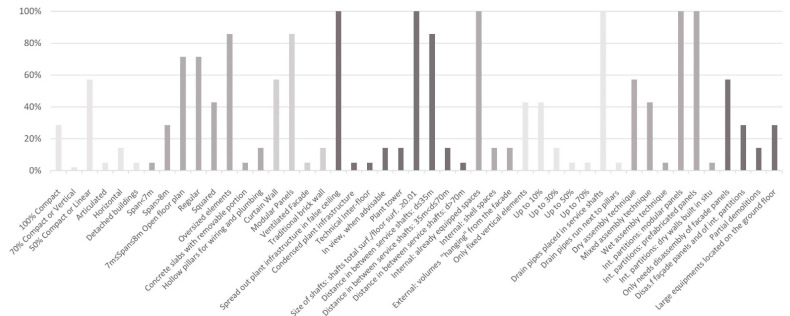
Histogram with percentage of evaluation parameters used. The horizontal x-axis lists the parameters included in the evaluation tool while on the vertical y-axes the fulfillment of each parameter based on the case studies analyzed is seen. The figure shows that some parameters are very common while others are rarer to be found in the selected case studies.

A comparative histogram based on the analytical framework applied to the case studies and our results conducted from the assessment tool for Open Building flexibility, are shown in Figure 3. The analysis demonstrates the most applied strategies (measurable variables) using the evaluation parameters for the selected healthcare facilities.

The most common type of shape parameters was 50% compact or linear, although the compact shape allows flexibility. The most applied strategy in the structure parameter is the oversized structural elements as it maximizes the building’s structural capacity to accommodate future expansion. Considering facade design, the most applied principle was having a modular facade and then comes the curtain wall system. For the building plant, the most applied strategies were spreading out plant infrastructure in false ceilings and minimizing the ratio of the total surface area of shafts-to-floor surface area to be less than 0.01. Internal equipped spaces are the most important and also the most applied in terms of expandability. As for restrictions, drainpipes placed in service shafts are the most applied strategy. Modular and prefabricated internal partitions are applied the most evenly though the dry assembly technique is a more fundamental strategy when it comes to technology parameters. The exchangeability of large equipment was achieved through one method, the disassembly of facade panels.

### 3.4. Optimized Assessment Tool

We conducted a critical review of the Open Building Assessment Tool (OBAT) in this section in order to understand the classification of the evaluation and analyses parameters, and the methods of evaluation and scoring for each parameter. We proposed modifications to each parameter, based on the critical review of the evaluation parameters our literature review. Each parameter is explained and a clear definition for each is generated with the assigned score according to its value in terms of flexibility for each healthcare facility.

#### 3.4.1. Critical Review of Evaluation Parameters

The original tool OBAT identified eight elements as evaluation parameters: shape, structure, facade, building plant, expandability, restrictions, technology, exchangeability of large equipment. Each of them is taken into consideration independently and is given an overall summary score with a range that varies between 0 and 10. Therefore, each of the indicators was assigned a value that expresses the degree of compliance with the flexibility characteristics of the Open Building framework for that specific aspect of the project.

(i) Shape parameter critical review

The main criterion for defining the efficiency of building shape/morphology is based on achieving flexibility, convenience, and cost-effectiveness, yet with the presence of significant ambiguity in the way the designers or users of the assessment tool would perceive such analysis parameters (100% compact, 70% compactor vertical, 50% compactor linear, articulated, horizontal, and detached buildings). As for compact classification, the main criterion for assigning scores is “compactness percentages”. What is meant by this term and the borderlines between each of the three identified compactness levels are not defined. In addition, the parameters merge different typological classifications such as 70% compact with vertical and 50% compact with linear. Although the vertical building gets a high score as the building plant’s main components are vertically stacked and placed in the shafts, how to determine verticality is not mentioned, and the same applies to the case of linearity. On the other hand, in the case of 50% compact, articulated, and detached classifications, there are no definitions.

(ii) Structure parameter critical review

The assessment tool aims to determine the flexibility of the building structure, regarding its capacity to accommodate extra loads concerning adding heavy medical equipment and/or vertical future expansion. The analysis parameter for structural spans is clearly defined and classified into three categories with a specific score each, which are (span < 7 m, span > 8 m and 7 m ≤ span ≤ 8 m), corresponding with the literature, healthcare design guidelines and selected case studies. While regular structural modulation is considered as an advantage in terms of flexibility, there should be tolerance as there might be some constraints that require breaking the regularity (i.e., site boundaries, spaces with special requirements, orientation, etc.). This tolerance should be also taken into consideration in the case of the squared analysis parameter, to avoid inflexible assessment that might negatively impact the total evaluation. As for the oversized elements, it should also consider the vertical expansion if needed in the future. The analysis parameter prefab slab “predalles” is not commonly used, and is not mentioned in the literature or the healthcare design guidelines.

(iii) Facade parameter critical review

The assessment tool’s main criteria for defining the efficiency of building facade is based on flexibility, neglecting the architectural articulation and technical aspects, yet without differentiating with intermediate categories between the completely opposite cases of the building being completely glazed “curtain wall”, and solid facades (masonry, bricks, etc.) with openings. Although curtain walls can allow total independence from the building structure, they neglect different cases of hybrid facades which combine solid and glazing with different proportions, causing uncertainties for designers and evaluators.

(iv) Building plant parameter critical review

The assessment tool’s main criteria for defining the efficiency of the building plant are capacity, distribution, and capability of adapting to future alterations or expansion. The analysis parameter of plant distribution is clearly defined and classified into three categories with specific score each, which are (spread out plant infrastructure in false ceiling, condensed plant infrastructure (varying height of false ceiling) and technical interfloor). The analysis parameters of distribution in raised floors and in view when advisable are both useful strategies in supporting functions such as laboratories and pharmacies. 

In the case of a plant tower, the mechanical floor should not be neglected considering its wide mention in literature and applied in the analyzed healthcare projects. Even though one of the main criteria for defining the building plant evaluation parameter is the capability to adapt to future hospital needs, the redundancy of the building plant is totally neglected. As for the distance between shafts analysis parameter, it is well defined based on the complexity of connections of the technical network and categorized into three classifications, and a score of (+4) is assigned to the first category as a distance less than 30 m creates a more efficient distribution of the service network. However, there are other elements that are equally important.

(v) Expandability parameter critical review

The assessment tool’s main criteria for defining the possibility of expanding the healthcare facilities is the availability of excess spaces to accommodate certain elements that facilitate the mass itself to expand within the existing structure. However, it neglects expandability through creating physical extensions which is a significant strategy for future-proofing of the facility. The expandability evaluation parameter is classified into two main categories, internal and external. Although the internal spaces are well defined, yet other equally important strategies are neglected, even though they are mentioned in literature and in healthcare design guidelines, such as providing soft spaces that can be retrofitted into service spaces, and open-ended corridors that allow horizontal expansion without sacrificing existing and functioning spaces. As for the internal expandability, only one external strategy is identified: volumes “hanging” from the facade, and a score of (+2) is assigned, even though it is not of great advantage when it comes to external expandability. We did not find other strategies for external flexibility.

(vi) Restrictions parameter critical review

The assessment tool’s main criteria for defining the structural restrictions that constrain future alterations of the healthcare facility is the percentage of the fixed vertical elements in the building plant that exist in several or all building floors. The restriction evaluation parameter is well defined and classified into five categories that are include only fixed vertical elements (connections and service shaft), up to 10%, up to 30%, up to 50%, up to 70%, respectively. With the lower percentage of fixed elements in the building, the more guaranteed is the level of space flexibility. Having minimal fixed elements is recommended. Although the classifications are well defined, the hierarchy of percentages and their respective scores are relatively unbalanced. Extra points are assigned to placement of draining techniques such as drainpipes placed in service shafts and drainpipes that run next to pillars. Since drainage pipes are considered complex elements of the hospital ward floors, combining them with either pillar or service shafts would significantly reduce the vertical constraints in the floor plans.

(vii) Technology parameter critical review

The assessment tool’s main criteria for defining the flexibility of building technology is the assembly and fabrication techniques of the interior walls/partitions. The analysis parameter of assembly techniques is well defined and classified into three categories according to the construction technique used that are: dry assembly technique, mixed assembly technique, and, wet assembly technique) respectively. Although internal partitions with specific analysis parameter characteristics such as modularity and embedded plant infrastructure have considerable scoring weight, other essential aspects such as movable/retractable partitions and using framed construction techniques are neglected, even though they are present in literature and the healthcare design guidelines.

(viii) Exchangeability of large equipment parameter critical review

The assessment tool’s main criterion for defining the possibility of exchanging large equipment because of technological and medical advancements in the future is carrying out the process with minimal intervention, no demolition, and in considerably short time. The analysis parameter of complexity of exchangeability of large equipment is well defined and classified into three categories according to the nature of intervention that is: only needs disassembly of facade panels, disassembly of facade panels and internal partitions, and partial demolitions, respectively. Although (+2) is assigned to large equipment located on the ground floor, it can miss the case of having the equipment located on a floor that is in direct contact with the outside. Considering future-proofing strategies, the equipment spaces should be designed with redundancy to accommodate equipment that may require extra storage areas.

#### 3.4.2. Modifications and Improvements of the Existing Tool

The parameter modifications are shown in Table 6 according to the critical review. After analyzing the evaluation parameters of the existing assessment tool and conducting an in-depth critical overview, we identified that all the defined parameters for evaluation are mainly concerned with the physical aspects of the healthcare facility with only a few minor ones addressing functional aspects.

We identified and extracted the flexibility principles related to functionality that play a vital role in guaranteeing future-proofing of the building based on the literature search, review of the healthcare design guidelines and a review of recent healthcare projects. Accordingly, a new evaluation parameter “functionality” offers a more comprehensive and precise evaluation for the healthcare facility, as seen in Table 8.

Functionality is an essential principle for the building, both from an efficiency and future-proofing points of view, since it considers adaptability, versatility, refit-ability, convertibility and expandability, as instrumental aspects that must be achieved. The functionality evaluation parameter contains six measurable variables: generic/universal rooms, space standardization, double function, overflow design, loose fit, furniture/equipment flexibility.

#### 3.4.3. Optimized Flexibility Assessment Tool

The assessment tool was designed to determine the degree of fulfillment of the essential principles of the flexibility concept. It was developed to evaluate flexibility in healthcare facilities during the design and planning phases and provides a control benchmark for the designer to enhance their proposals. Applying it to existing facilities helps to better appreciate to what extent the building satisfies the criteria, concepts of flexibility and hence what needs to be modified if needed.

The new tool consists of nine evaluation parameters, with each parameter subdivided into measurable variables with a score range varying between 0 and 10 (see Text S1 for the full list and scoring). Each evaluation parameter achieves a specific score that reflects the level of application of flexibility principles. Five score ranges were identified using this weighing system, and which correspond to five different levels of compliance with the flexibility criteria, thus helping to determine to what extent the building is flexible.

### 3.5. Results of Comparison between Original and Optimized Tools

We tested the revised tool on two selected healthcare facilities from the previously evaluated case studies (e.g., Hospital Südspidol and Aarhus Hospital). We checked the viability of the optimized tool and simultaneously compared the scores of each evaluation parameter using both versions of the original and revised tools.

For each of the two case studies, we compiled results from the optimized assessment generated along with a critical comparison with the results from the original assessment. We drew diagrams to recap the results of the final score from the optimized assessment tool and compared them to see to what extent each evaluation parameter was applied. We also classified the building according to its assessment score and to determine whether it satisfied the criteria for an Open Building and to what extent.

#### Case Study 1 (CS1)

The new assessment tool was applied to the healthcare facility CS1 and the total assigned score was 75% (64/85), hence it was classified as a flexible building, but with some aspects to be improved. The final results show that the total assigned score from the original assessment tool was 72%, and 75%, using the modified tool.

#### Case Study 2 (CS2)

The new assessment tool was applied to the healthcare facility and the total assigned score was 73% (63/86), hence it was classified as a flexible building, but with some aspects to be improved. The final results show that the total assigned scores from the original assessment tool were 69% and 73%, using the modified one. A detailed comparison between the two cases is provided in Table 9.

**Figure 4:**
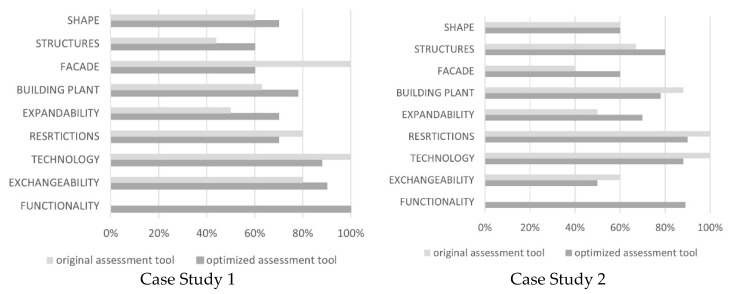
Percentage of application of evaluation parameters of original (OBAT) and optimized assessment tools (OFAT).

Figure 4 illustrates the results with the total assigned scores, and indicates to what extent each one of the evaluation parameters was fulfilled in the facility classification.

## 4. Discussion

### 4.1. The Importance of Flexibility Evaluation

Healthcare facilities are complex structures with a mixture of social, cultural, economic, technological, and architectural aspects. For healthcare facilities to fulfill their roles, it is essential that they should be planned and designed for the present and the future. Hospitals should be flexible to changing needs. John Weeks in 1954 highlighted the importance of multifunctional potential for interconnecting examination and consulting rooms and how the workflow of nurses can be modified in the interests of improved patient care [47]. It was a model for flexibility within a logical plan, a large building that was serviced and flexible which he called, “indeterminate architecture”. This approach allows for infinite changes to accommodate different workflow needs and new technology innovations.

While emphasizing the importance of the building’s flexibility it is important to highlight other areas of improvement. Several alterations can indeed be carried out on the operational level of the facility without any change at the physical level while other changes are made by adapting the users to maximize their experience of the given environment. It is necessary to evaluate how such changes would affect the overall operation and performance of the facility and impact on the provider’s workflow. Hospital buildings should have the capability to accommodate alteration of functions and not just designed rigidly to serve a specific purpose. In fact, such approaches can only be addressed if a participatory design and multidisciplinary cooperation between different fields, disciplines and professions is in place [48,49]. The needs of different stakeholders and users can be met in a flexible physical built environment that accommodates different activities such as when designing cancer care facilities [50].

Our multi-method research approach addressed both contemporary and innovative approaches in healthcare design that are divided into multiple levels which are not only physical but also operational, such as architectural, structural, engineering, technological and functional levels. Flexibility is the essential principle in contemporary and futuristic hospitals, and designers must prepare for changing needs. Implementing an easy-to-use tool applicable to different design stages can improve the effectiveness of project flexibility, both in the product and process dimensions, by reducing the cost of unplanned changes during advanced construction, refurbishment and operation phases [51,52,53].

### 4.2. The Optimization of the Tool

The flexibility principle is not commonly considered nor used by practitioners, however it is used in various hospitals worldwide without intentionally aiming for flexibility [20,49]. We demonstrated using the original assessment tool that only one in seven of the most advanced healthcare facilities scored higher than 80%. It was evident that there is a significant level of ambiguity and hence uncertainty during the evaluation process which leads to inaccurate and/or misleading results and undermines future reuse opportunities. This considerably affects the total assigned score and the categorization of the facility.

The new assessment tool (OFAT) is designed to improve the degree of fulfillment of the essential flexibility principles including the Open Building concept both in design and operation phases. We found that, in two case studies, applying the OFAT led to a more comprehensive assessment when compared to the old tool.

### 4.3. The Application of OFAT Regarding COVID-19

COVID-19 affected all segments of the health care industry; some effects will be temporary, but others are likely to have profound long-term consequences [54], such as the reorganization and capacity of hospitals [55,56]. The characteristics of health care systems, high levels of uncertainty and changing technology and treatment methods, are also driving the need for enhanced flexibility [57]. Facilities resilience and space planning flexibility remain two of the most important challenges for hospital design.

We optimized the evaluation tool, OFAT, to provide a benchmark and monitoring system for healthcare designers to improve their proposed flexibility during the design and planning phases, as well as being applicable for existing facility designs. The COVID-19 pandemic has required health care systems to undergo a paradigm shift and be prepared for new emergencies [58]. Implementation of a new assessment tool (OFAT) responds to this demand and enhances the ability of healthcare facilities to meet challenges in the future. It opens up possibilities for evaluation of the adequacy of hospital structures to new flexibility challenges in light of new investments initiatives [59].

### 4.4. Limitations and Future Developments

Firstly, as mentioned above one of the limitations encountered in the design guidelines review process was the unavailability of a free version of the USA design guidelines (FGI). Secondly, the limited application to two pilot case studies underscores the need to further expand the testing phase. Further study is needed to consider the potential effects of applying the assessment tool to a wider sample of facilities and geographic locations.

## 5. Conclusions

We used a five-step research methodology that included a literature review, review of design guidelines, case studies evaluation, improved assessment tool, and a revised final tool test, the new Optimized Flexibility Assessment Tool (OFAT). The OFAT is well suited and robust for assessing the flexibility of healthcare facilities. Increasing the flexibility and adaptability of physical structures and services, both at the levels of facility itself and its network, are urgently needed. Ensuring rapid, efficient, and high-quality interventions are needed even under exceptional conditions (with a focus on the flexibility and environmental quality of the spaces which, in addition to supporting the health response, is reflected in perceived quality on patients and operators).

While our results are exploratory and further long-term research is needed, our findings resulted in the development of the OFAT assessment tool, OFAT, to evaluate the extent to which a healthcare facility meets the principles of flexibility.

## Figures and Tables

**Table 1:** Searching rules and selecting criteria for the literature review.

Searching Rules	Selection Criteria	Outcomes
(“FLEXIBILITY IN HEALTHCARE FACILITIES” OR “FLEXIBLE HOSPITAL” OR “FLEXIBILITY PRINCIPLES IN HEALTHCARE”) AND (“ADAPTABILITY” OR “STANDARDIZATION” OR “LEVELS OF FLEXIBILITY” OR “IMPACT OF FLEXIBILITY”)	Focus on healthcare facilities and design English language Definition of clear design strategies Peer-reviewed journal Published after 2000	28 references about the principles and strategies of flexibility (full list in Table S1)

**Table 2:** Searching rules and selection criteria for review of healthcare design guidelines.

Searching Rules	Selection Criteria	Outcomes
“HEALTHCARE DESIGN GUIDELINES” OR “HEALTH BUILDING NOTES” OR “HEALTHCARE FACILITIES LEGISLATION” OR “HOSPITAL PLANNING GUIDELINES”	Developed by the leading countries in the field of healthcare design English language Released after 2010 Applied considerable flexibility strategies and/or design principles in the guidelines	Five healthcare design guidelines

**Table 3:** Searching rules and selection criteria for case studies.

Searching Rules	Selection Criteria	Outcomes
“INNOVATIVE HEALTHCARE FACILITY” OR “FLEXIBLE HOSPITALS” OR “OPEN BUILDING IN HEALTHCARE” OR “ADAPTABLE HOSPITALS”	Considered as best practices in the design community Diversity in geographical locations/contexts Designed after 2000 Diverse in scale (standalone building or complex) Detailed and relevant data is accessible to researchers	Seven case studies

**Table 4:** Levels and types of Flexibility [21].

Hospital Facility Scale	Explanation
Hospital complex	Combination of all the buildings and external spaces which define the healthcare facility as a whole
Building	Individual building identifiable within the broader system; in the case of healthcare facilities made up of an individual single-block building, this level will have many features in common with the hospital complex
Functional unit	Combination of rooms grouped by similarity of functions, for example, wards, surgical block, central heating plant, etc.
Individual room	Individual space confined and delimited by walls, identifiable individually within a functional unit such as a room in a ward, a doctor’s consulting room, etc.
**Types of Flexibility**	
Constant surface flexibility	Facility should be able to develop without reformation of overall surface area (GFA), adapting to alterations to its spatial organization due to development of demand, innovations in medical science, or the redevelopment of functions. In this level high importance is dedicated to layout planning and space management capacity [45]
Variable surface flexibility	Facility, based on the demands, should be able to accommodate scalability in terms of expansion or reduction without creating any disturbance or obstruction for the facility activities
Operational flexibility	Functions of the hospital should be able to adjust and adapt in order to enhance its operation through alterations of different services

**Table 5:** Flexibility matrix for healthcare facilities [21].

Levels of Flexibility	Types of Flexibility	Typological-Spatial Strategies
Hospital complex	Constant surface flexibility	Flexibility of access systems
		Functional flexibility of the system
		Reuse of the hospital complex
		Redundancy of space for plant
	Variable surface flexibility	Existence of unused building land
		Strategies for increasing the volume of individual buildings
	Operational flexibility	Modular, replaceable, and maintainable plant
		Presence of networked information systems
		The use of building automation and control systems (for overall management)
		The use of flexible contractual/financial arrangements
		Outsourcing of support services
Building	Constant surface flexibility	Existence of shell space for expansion
		Structural flexibility
		Oversizing of load-bearing structures
		Modifiability of the envelope
		Presence of spaces for building plant infrastructure
		Flexibility and automation of segregated pedestrian routes
	Variable surface flexibility	Oversizing of load-bearing structures
		The use of blank facades
		Possibility of modular expansion
		Tiered building
Functional Unit	Operational flexibility	Modular, replaceable, and maintainable plant
		The use of building automation and control systems (at a building level)
		Efficient programmed maintenance
		Life cycle cost
	Constant surface flexibility	The use of internal dry partitions
		The use of movable internal walls and walls with wall-mounted fittings
		The use of movable internal partitions
		Presence of spaces for service building infrastructure
	Variable surface flexibility	Possibility of extending the entire functional unit upwards/sideways
		Presence of verandas/setbacks
	Operational flexibility	Plan with the flexibility of use
Individual Room	Constant surface flexibility	Functional flexibility of the room
	Variable surface flexibility	The possibility of extensions upwards/sideways
	Flexibility of use	Providing for multifunctional rooms
		Plant for multifunctionality
		Information systems services for multifunctionality
	Adaptivity to the user	The use of movable furniture and vertical screening
		Customizable humanization of the room

**Table 6:** Flexibility principles matrix for healthcare guidelines.

Healthcare Design Guidelines	Planning Models	Adaptability	Convertibility	Expandability	Standardization	Modular Design	Open Building Concept	Room Utilization	Unit Planning	Open Planning	Generic Spaces	Loose Fit	Accessibility	Circulation	Circulation Core	Masterplan Flexibility	Overflow Design	Prefab. Internal Elements	Structural Loading Capacity	Floor Structure Flexibility	Infrastructure Capacity	Sustainability	Construction Flexibility	Power Plant	Furniture Flexibility	Equipment Flexibility	Interstitial Floor	Ceiling Height	Facade Design
U.K. (DH Health Building Notes)	•	•	•	•	•	•	•	•	•		•	•	•	•	•	•	•	•	•		•		•	•	•	•			
Australia (Australian Healthcare Facility Guidelines)	•	•		•	•	•		•		•	•		•	•	•		•				•	•		•	•		•	•	•
Canadian (Canadian Healthcare Facilities)	•	•	•	•	•	•	•	•		•		•	•		•		•			•	•				•				
The international guidelines (authored by Total Alliance Health Partners International (TAHPI)	•	•		•	•	•		•	•			•	•	•			•												

A corresponding “•” within the matrix indicates that a Healthcare Design Guideline addresses a specific flexibility principle.

**Table 7:** Evaluation Parameters Scores of Selected Case Studies.

Evaluation Parameters	CS 1	CS 2	CS 3	CS 4	CS 5	CS 6	CS 7
Shape	6/10	10/10	2/10	10/10	6/10	6/10	6/10
Structure	4/9	5/9	7/9	7/9	4/9	6/9	6/9
Facade	10/10	10/10	0/10	10/10	10/10	4/10	4/10
Building Plant	5/8	7/8	7/8	9/9	7/8	7/8	8/9
Expandability	5/10	5/10	5/10	8/10	5/10	5/10	7/10
Restrictions	8/10	6/10	10/10	8/10	8/10	10/10	10/10
Technology	8/8	6/8	6/8	6/8	8/8	8/8	8/8
Exchangeability	8/10	8/10	2/10	4/10	8/10	6/10	8/10
Summary	54/75	57/75	39/75	62/76	56/75	52/75	57/76
Total score ^1^	72.0%	76.0%	52.0%	81.5%	74.6%	69.3%	75.0%

^1^ 0 to 20%: not an Open Building; 21% to 40%: following some principles, but it cannot be considered an Open Building; 41% to 60%: following several principles of the Open Building approach; 61% to 80%: it can be considered an Open Building but with some aspects to be improved; 81% to 100%: a model of Open Building.

**Table 8:** Proposed modifications for each evaluation parameter.

Evaluation Parameter	Modifications
Shape	Merged and not correlated morphological classifications of “70% compact with vertical” and “50% compact with linear” are to be split into different classifications.
	As a result of splitting the merged classifications, the number of analysis parameters increases from six to eight for well-defined and more accurate evaluation. Consequently, the scores are reconsidered to match the new modifications.
Structure	A tolerance of 20% is added to the regular grid analysis parameter to avoid inflexible assessment that might negatively impact the total evaluation. Consequently, the (+1) is assigned to 80% to 100% regular grid instead.
	The former case is also applied to the squared grid analysis parameter, applying a tolerance of 20%. Hence, the (+1) is assigned to 80% to 100% squared grid instead.
	The oversized structural elements are to be redefined to embrace not only the capacity of the structure to accommodate extra medical equipment, but also the vertical expansion of the building if needed. Regarding vertical expansion, there is no definitive percentage of oversizing, but it will depend on each individual case according to building height legislation of the project location.
	The analysis parameter “predalles” is excluded from the assessment, as it is not an instrumental technique when it comes to structural flexibility.
	A new analysis parameter “ceiling height ≥ 4 m” is added, as it has an essential impact on the flexibility of the healthcare facility to enable future convertibility according to the literature and healthcare design guidelines. It is assigned (+1) score.
Facade	The curtain wall analysis parameter is redefined into three different classifications that are 100% curtain wall, 75% curtain wall and 50% curtain wall, and their scores are (+6), (+4) and (+2) respectively.
	As a result of redefining the curtain wall analysis parameter, the number of analysis parameters increases from four to six for more accurate evaluation.
Building Plant	The analysis parameters “distribution in raised floor” and “in view when advisable” (modified to: exposed installations, when required), are to be merged in one analysis parameter as they are considered different techniques that serve the same purpose.
	A new analysis parameter “mechanical floor” is added, as it facilitates the free transition between functions (for instance: bed tower and operational block) that have different spatial organization and technical/structural requirements. Hence, (+1) score is assigned.
	Distances between shafts maximum score are reduced from (+4) to (+2). Even though it plays an essential role in providing the necessary flexibility to building plant. However, there are other elements that have no less importance.
	A new analysis parameter “redundancy of building plant” is added, as it enables to accommodate future alterations and additions to the building, according to the literature and design guidelines. It is assigned (+2) score.
Expandability	A new analysis parameter “open-ended corridor and/or large spaces on building’s end” is added, as it allows horizontal expansion without the need to remove the spaces of building ends and disturbing the ongoing functions. Hence, (+1) score is assigned.
	Another new analysis parameter “soft spaces: to be retrofitted into service spaces if needed” is added, as it maximizes the capability of the building to respond to functional future needs. In this case, (+1) score is assigned.
	Internal: already equipped spaces score reduced from (+5) to (+4), internal: shell spaces also is reduced from (+3) to (+2).
	Another new analysis parameter “availability of neighboring plot” is added, since it guarantees the possibility of physical expansion. In this case, (+1) score is assigned.
	“External: volumes ‘hanging’ from the façade” score is to be evaluated according to the third evaluation method “alternative points” instead of the second evaluation method. In this case, (+1) score is assigned.
Restrictions	The “percentage of fixed elements” analysis parameter is classified into four categories instead of five which are only fixed vertical elements (connections and service shaft), fixed elements of building plant: up to 25%, fixed elements of building plant: up to 50% and fixed elements of building plant: up to 75%.
	As a result of the reclassification of the former analysis parameter, the scoring of each parameter is updated to (+6), (+4), (+2) and (zero) respectively for more accurate evaluation.
	The analysis parameters “drain pipes placed in service shafts” and “drain pipes run next to pillars” are to be merged into one analysis parameter as they are considered different techniques that serve the same purpose. Consequently, the same score of (+1) is assigned.
	A new analysis parameter “adjustability of service shafts” is added, since their adjustability maximizes the capability of the building to respond to alterations in technical and clinical requirements. In this case, a score of (+2) is assigned.
	A new analysis parameter “grouped vertical circulation elements” is added, as it maximizes the future planning so that the rest of the floor space is contiguous and open. Hence a score of (+1) is assigned.
Technology	A new analysis parameter “internal partitions: movable/retractable” is added since it guarantees that spaces can be adjusted by just moving elements. They allow various flexible ways for the usage of space by changing the communication degree between neighboring rooms. In this case, a score of (+1) is assigned.
	A new analysis parameter “internal partitions: framed construction” is added, due to allowing partition walls to be altered in case of maintenance or necessity of change. Hence, a score of (+1) is assigned.
	As a result of adding two new analysis parameters, the scoring of “internal partitions: modular panels” and “internal partitions: panels set up with plant infrastructure” is reduced from (+2) to (+1).
Exchangeability	A new analysis parameter “equipment spaces with redundancy” is added, since it guarantees that spaces can be adapted to future requirements and accommodate new equipment. In this case, a score of (+1) is assigned.
	As a result of adding the former analysis parameter, the score of “large equipment in ground floor” is updated to (+1). Also, this parameter is redefined to include “equipment in floor with direct contact to outside”.
Functionality ^1^	The highest score (+4) is assigned when having generic/universal rooms since it supports resisting unnecessary variation in similar components, where the change in functionality can be accommodated in one standard design.
	A lower score of (+2) is assigned to the presence of space standardization, which is accredited to definition, specification, quality, and reduction errors due to repeatedly in addition to allowing adapting to future transformation and demands of the users of the facility.
	Double function is assigned a score of (+1), as it allows changes in operation mode through sharing of space.
	Overflow design is assigned a score of (+1) because it maximizes the capability of the space to accommodate multiple functions that do not have crossing time schedules. It is very beneficial in case of disasters.
	While loose fit is assigned a score of (+1) because it is a principle in which spaces adequately respond to today’s operational policy and have the inherent flexibility to adapt to a range of alternatives.
	In furniture/equipment flexibility, fulfilling either one or both get a score of (+1) since they permit movement into different areas for flexibility of function.

^1^ A new evaluation parameter “functionality” was added in order to provide a more comprehensive and precise evaluation of healthcare facilities.

**Table 9:** Detail of OFAT application on Case Study 1 and Case Study 2.

Parameter	Case Study 1	Case Study 2
Shape	7/10 (70%)	6/10 (60%)
Structure	6/10 (60%)	8/10 (80%)
Facade	6/10 (60%)	6/10 (60%)
Building Plant	7/9 (78%)	7/9 (78%)
Expandability	7/10 (70%)	7/10 (70%)
Structural Constraints	7/10 (70%)	9/10 (90%)
Technology:	7/8 (88%)	7/8 (88%)
Exchange of Large Equipment	9/10 (90%)	5/10 (50%)
Functionality	8/8 (100%)	8/9 (89%)
Total	64/85 (75%)	63/86 (73%)

## Data Availability

The data presented in this study are available in the present article and in its Supplementary Materials reported above.

## Supplementary Materials

The following are available online at https://www.mdpi.com/article/10.3390/ijerph182111478/s1, Table S1: Full List of 28 Papers Collected, Text S1: Assessment Tool and Scoring Criteria.
