# Supplementary material for: Flexibility during the COVID-19 Pandemic Response: Healthcare Facility Assessment Tools for Resilient Evaluation
Source: Int J Environ Res Public Health. 2021 Oct 31;18(21):11478. doi: 10.3390/ijerph182111478 (PMC8583089; doi:10.3390/ijerph182111478)
Supplement: Supplementary file 1 [file ijerph-18-11478-s001.zip › ijerph-1384060-supplementary/Table S1.pdf]

n°	Authors	Title	Year	Source	Before/After COVID-19 outbreak
1	Smolova M., Smolova D.	**Emergency architecture. Modular construction of healthcare facilities as a response to pandemic outbreak**	2021	E3S Web of Conferences	After
2	Pilosof N.P.	**Building for Change: Comparative Case Study of Hospital Architecture**	2021	Health Environments Research and Design Journal	After
3	Marinelli M.	**Emergency Healthcare Facilities: Managing Design in a Post Covid-19 World**	2020	IEEE Engineering Management Review	After
4	Halberthal M., Berger G., Hussein K., Reisner S., Mekel M., Horowitz N.A., Shachor-Meyouhas Y., Geffen Y., [illegible]	**Israeli underground hospital conversion for treating COVID-19 patients**	2020	American journal of disaster medicine	After
5	Brown D.R., Hennecke P., Nottebrock D., Dhillon P.	**Vancouver Convention Health Centre (COVID-19 Response): Planning, implementation, and four lessons learned**	2020	American journal of disaster medicine	After
6	Capolongo S., Gola M., Brambilla A., Morganti A., Mosca E.I., Barach P.	**COVID-19 and healthcare facilities: A decalogue of design strategies for resilient hospitals**	2020	Acta Biomedica	After
7	Lavikka R.H., Kyrö R., Peltokorpi A., Särkilahti A.	**Revealing change dynamics in hospital construction projects**	2019	Engineering, Construction and Architectural Management	Before
8	Prugsiganont S., Jensen P.A.	**Identification of space management problems in public hospitals: The case of Maharaj Chiang Mai Hospital**	2019	Facilities	Before
9	Sicignano E., Petti L., Di Ruocco G., Scarpitta N.	**A model flexible design for pediatric hospital**	2018	Lecture Notes in Civil Engineering	Before
10	Maben J., Griffiths P., Penfold C., Simon M., Anderson J.E., Robert G., Pizzo E., Hughes J., Murrells T., [illegible]	**One size fits all? Mixed methods evaluation of the impact of 100% single-room accommodation on staff and patient [illegible]**	2016	BMJ Quality and Safety	Before
11	Capolongo S., Buffoli M., Nachiero D., Tognolo C., Zanchi E., Gola M.	**Open building and flexibility in healthcare: Strategies for shaping spaces for social aspects**	2016	Annali dell'Istituto Superiore di Sanita	Before
12	van der Zwart J., van der Voordt T.J.M.	**Adding Value by Hospital Real Estate: An Exploration of Dutch Practice**	2016	Health Environments Research and Design Journal	Before
13	Astley P., Capolongo S., Gola M., Tartaglia A.	**Operative and design adaptability in healthcare facilities [Adattabilità operativa e progettuale nelle strutture [illegible]**	2015	TECHNE	Before
14	Del Gatto M.L., Morena M., Truppi T.	**Organizational models for the flexible management of hospitals [Modelli organizzativi per la flessibilità gestionale [illegible]**	2015	TECHNE	Before
26	Ahmad, A.M.; Price, A.; Demian, P.	**Impact of Space Flexibility and Standardisation on Healthcare Delivery**	2014	International Journal of Applied Science and Technology	Before
15	Browne E.M.	**Redesigning and retrofitting existing facilities for behavioral healthcare.**	2013	Journal of healthcare protection management : publication of the International Association for Hospital Security	Before
16	Ebrahimi A., Mardomi K., Rahimabad K.H.	**Architecture capabilities to improve healthcare environments**	2013	Trauma Monthly	Before
17	Wilson G., Kishk M.	**Key criteria of sustainable hospital refurbishment: A stakeholder review**	2012	Association of Researchers in Construction Management, ARCOM 2012 - Proceedings of the 28th Annual [illegible]	Before
18	Carthey J., Chow V., Jung Y.-M., Mills S.	**Flexibility: Beyond the buzzword-practical findings from a systematic literature review**	2011	Health Environments Research and Design Journal	Before
25	Ahmad, M.A.; Price, A.D.F.; Demian, P.; Lu, J.	**Space Standardisation and Flexibility on Healthcare Refurbishment.**	2011	Architecture in the Fourth Dimension	Before
28	Olsson, N.O. And Hansen, G.K.,	**Identification of Critical Factors Affecting Flexibility In Hospital Construction Projects**	2010	Herd: Health Environments Research & Design Journal	Before
21	Bjørberg, S.; Verweij, M.	**Life-Cycle Economics: Cost, Functionality and Adaptability. Investing in hospitals of the future.**	2009	Copenhagen: European Observatory on Health Systems and Policies	Before
27	De Neufville, R., Lee, Y.S. and Scholtes, S.	**Using Flexibility to Improve Value-For-Money in Hospital Infrastructure Investments**	2008	Infrastructure Systems and Services: Building Networks for A Brighter Future (Infra), 2008 First International [illegible]	Before
20	Pati, D.; Harvey, T.; Cason, C.	**Inpatient Unit Flexibility: Design Characteristics of a Successful Flexible Unit**	2008	Environment and Behavior	Before
24	Reiling, J.	**Safe design of healthcare facilities**	2006	Qual Saf Health Care.	Before
23	Hendrich, A.L.; Fay, J.; Sorrells, A.K.	**Effects of Acuity-Adaptable Rooms on Flow of Patients and Delivery of Care**	2004	American Journal of Critical Care	Before
19	Reiling J.G., Knutzen B.L., Wallen T.K., McCullough S., Miller R., Chernos S.	**Enhancing the traditional hospital design process: a focus on patient safety.**	2004	Joint Commission journal on quality and safety	Before
22	Gallant, D.; Lanning, K.	**Streamlining Patient Care Processes through Flexible Room and Equipment Design**	2001	Critical Care Nursing Quarterly	Before
