# Supplementary material for: Flexibility during the COVID-19 Pandemic Response: Healthcare Facility Assessment Tools for Resilient Evaluation
Source: Int J Environ Res Public Health. 2021 Oct 31;18(21):11478. doi: 10.3390/ijerph182111478 (PMC8583089; doi:10.3390/ijerph182111478)
Supplement: Supplementary file 1 [file ijerph-18-11478-s001.zip › ijerph-1384060-supplementary/Text S1.pdf]

OFAT

Optimized Flexibility Assessment Tool for Hospital Planners

This Assessment Tool is designed and optimized to determine the degree of fulfillment of the essential principles of Flexibility concept. It is developed to evaluate flexibility in healthcare facilities during designing and planning phase that provides a sort of a control system for the designer to enhance their proposals. And also can be applied on existing ones to understand to what extent the building satisfies the criteria, concepts of Flexibility and hence what needs to be modified if needed.

The Assessment Tool consists of 9 evaluation parameters, each one is subdivided into certain analysis parameters upon which a score with a range varying between 0 and 10 is assigned. Hence, each evaluation parameter will be assigned a specific score that reflects the level of application of flexibility principles. Using this weighing system, five score ranges are identified which correspond to five different levels of compliance with the Flexibility criteria. Thus, determining to what extent the building is flexible. Attached with the assessment tool a Glossary of all evaluation and analysis parameters.

Assessment Criteria & Glossary

After optimizing the original assessment tool, the newly optimized tool evaluates flexibility as it is a broader concept that includes Open Building concept.

The final outcome of the assessment tool is categorized into five different ranges:

- 0 to 20%: definitely not a Flexible Building
- 21% to 40%: following some principles, but it cannot be considered a Flexible Building
- 41% to 60%: following several principles of the Flexible Building approach
- 61% to 80%: it can be considered a Flexible Building but with some aspects to be improved
- 81% to 100%: model of Flexible Building

Assessment Tool Scales - Shape

Evaluation Parameters	Analysis Parameters	Score	Assigned Score
Shape	100% Compact	10	
	75% Compact	9	
	Vertical	8	
	50% Compact	7	
	Linear	6	
	Articulated	4	
	Horizontal	2	
	Detached	0	
Shape Total Assigned Score			/10

Criteria

Assuming that the generic building mass is a perfect cube of XYZ, as X "length", Y "width" & Z "height".

1. 100% Compact: In this case, all mass edges XYZ are equal with tolerance: +/- 20% at any direction.

2. 75% Compact: In this case, the height ranges from 1.2Z to 2Z, provided that X & Y do not exceed 1.2X & 1.2Y.

3. Vertical: In this case, the height (Z) of the building mass should at least exceed double the length of the longest base edge (X or Y).

4. 50% Compact: In this case, the base sides ranges from 1.2X to 2X or 1.2Y to 2Y, provided that (Z) does not exceed 1.2Z.

5. Linear: In this case, the length (X) or the width (Y) exceeds double the height (Z) of the mass.

6. Articulated: In this case, the masses are irregular and intersecting with different morphologies and heights.

7. Horizontal: In this case, the height (Z) less than the length of the shortest edge (X or Y).

8. Detached: In this case, the complex consists of completely separate masses without any internal links.

Note:In the case of separate 100% Compact masses yet only linked with bridges, the evaluation of the entire complex would be considered either 50% Compact or Linear, depending on the combined morphology of the masses. As they would be evaluated as one entity, but not considered as a single compact building, since inevitably each mass would require its own plant and mechanical installations.

Assessment Tool Scales - Structure

Evaluation Parameters	Analysis Parameters	Score	Assigned Score
Structure	7m ≤ Span ≤ 8m	4	
	Span > 8m	2	
	Span < 7m	1	
	Regular Grid	+1	
	Squared Grid	+1	
	Oversized Structure Elements	+1	
	Punch Through Concrete Slabs for Vertical Circulation	+1	
	Hollow Pillars for Technical Installations	+1	
	Ceiling Height ⩾ 4m	+1	
Structure Total Assigned Score			/10

Criteria

1. Structural Spans: The 3 different structural spans are to be measured from the axis/center of the pillars.

2. Regular Grid: A fixed grid modulation that is arrayed throughout, bearing in mind there is a tolerance of 20%.

3. Squared Grid: A squarish grid modulation that is arrayed throughout, bearing in mind there is a tolerance of 20%.

4. Oversized Structure Elements: Increasing the capacity of structural elements to sustain extra loads, bearing in mind there is a tolerance, that depends on each individual case according to buildings' heights legislation of the project location

5. Punch Through Concrete Slabs for Vertical Circulation: A squarish area in the concrete slab but with no reinforcement that allows adding vertical circulation elements, shafts, mechanical installations, etc.

6. Hollow Pillars for Technical Installations: Hollow columns that act as shafts for wiring and plumbing to pass through them.

7. Ceiling Height: an instrumental element when it comes to maximizing possibility to future flexibility, for instance converting patient rooms into operation room.

Assessment Tool Scales - Facade

Evaluation Parameters	Analysis Parameters	Score	Assigned Score
Facade	100% Curtain Wall	6	
	75% Curtain Wall	4	
	50% Curtain Wall	2	
	Ventilated Facade	0	
	Traditional Masonry/Brick Wall	0	
	Modular Panels	+4	
Facade Total Assigned Score			/10

Criteria

1. Curtain Wall: The 3 different curtain wall categories are classified according to glazing to solid percentage and to their independence from the building structure.

2. Ventilated Facade: A system that allows the formation of an air chamber between the external wall of the building and the cladding.

3. Traditional Masonry/Brick Wall: Traditional Masonry/Brick Wall has significant limitations as demolition is essential in case intervention, that interrupts the on going functions of the facility.

4. Modular Panels: Modulated glazing or cladding panels that can be easily assembled or disassembled.

Note: In some cases, specific functions necessitates to have solid facades, such as (technical floor, interstitial floor, auditorium, etc.). These solid portions are to be neglected from the calculation the percentage of the Curtain Wall in the building.

Assessment Tool Scales – Building Plant

Evaluation Parameters	Analysis Parameters	Score	Assigned Score
Building Plant	Distance in between Service Shafts: d ≤ 35m	2	
	Distance in between Service Shafts: 35m < d ≤ 70m	1	
	Distance in between Service Shafts: d > 70m	0	
	Redundancy for Building Plant	+2	
	Distribution in Raised Floor and/or Exposed Installations if Required	+1	
	Plant Tower	+1	
	Technical Floor	+1	
	Service Shafts Area: Shafts Total Surface/Floor surface ⩾ 0.01	+1	
	Spread out Plant Infrastructure in False Ceiling*	+2*	
	Condensed Plant Infrastructure (Varying Height of False Ceiling)*	+1*	
	Technical Interstitial Floor*	+1*	
Building Plant Total Assigned Score			/10

Criteria

1. Distance between Service Shafts: The 3 different distances between shafts are identified according to their service network integration efficiency.

2. Redundancy for Building Plant: Building Plant to be designed with extra spaces in order to be adaptable and accessible through time, planned and constructed in way that can accommodate future alterations and addition.

3. Distr. in Raised Floor and/or Exposed Installations if Required: As for Raised Floor, it is an elevated structural floor that creates continuous void under the floor surface that can be utilized to route all technical systems. It is useful in laboratories and offices. While Exposed Installations When Required, is a technique in which technical outputs are visible and accessible especially in patient rooms that enhances functional flexibility of space.

4. Plant Tower: A vertically compacted service plant from which service network branches horizontally for floor occurs.

5. Mechanical Floor: It is a story designed especially in vertical/compact buildings that is dedicated to accommodate the mechanical equipment of the floors above it, emphasizing the functional layout of the building by dividing it into different blocks.

6. Service Shafts Area: According to the calculations of area of the service shafts, with tolerance for future needs, the shafts total area/floor area should be equal to or greater than 1%.

7. Distribution of Plant in Ceiling/Interstitial Floor: The 3 different distribution techniques whether in ceiling or in a dedicated floor "Interstitial Floor".

Assessment Tool Scales – Expandability

Evaluation Parameters	Analysis Parameters	Score	Assigned Score
Expandability	Internal: Already Equipped Spaces	+4	
	Internal: Shell Spaces	+2	
	Open Ended Corridor and/or Large Spaces on Building's End	+1	
	Soft Spaces: to be Retrofitted into Service Spaces if needed	+1	
	External: Volumes "Hanging" from the Facade	+1	
	Availability of Neighboring Plot	+1	
Expandability Total Assigned Score			/10

Criteria

1. Internal: Already Equipped Spaces: Spaces designed and constructed with the different service networks that necessitates placing required internal partitions and furniture, to be used when needed.

2. Internal: Shell Spaces: Already constructed spaces that need more time and invasive technical operations, such as the installation of plant networks to be used when needed.

3. Open-Ended Corridor and/or Large Spaces on Building' s End: Designing circulation paths (corridors) that are open ended (without dead ends), in order to guarantee the possibility of healthcare zones to expand or retract.

4. Soft Spaces: to be Retrofitted into Service Spaces if needed: Extra space where backup facilities can be accommodated in case of need or disaster or damage. Providing soft spaces next to technical services is preferred as it may be retrofitted into service spaces to meet the future services needs with no interruption to the on going healthcare facility functions.

5. External: Volumes "Hanging" from the Facade: Extruded masses from the facade which are included in the structure calculations of the facility, hence it does not need extra supporting structure in case of expansion.

6. Availability of Neighboring Plot: Extra adjacent areas that lies within the property borders that facilitates horizontal expansion if need in the future. This maximizes operational flexibility.

Assessment Tool Scales – Structural Constraints

Evaluation Parameters	Analysis Parameters	Score	Assigned Score
Structural Constraints	Only Fixed Vertical Elements (Circulation & Service Shafts)	6	
	Fixed Elements of Building Plant: Up to 25%	4	
	Fixed Elements of Building Plant: Up to 50%	2	
	Fixed Elements of Building Plant: Up to 75%	0	
	Drain Pipes: In Service Shafts or Running Next to Pillars	+2	
	Adjustability of Service Shafts	+1	
	Grouped Vertical Circulation Elements	+1	
Structural Constraints Total Assigned Score			/10

Criteria

1. Fixed Elements: The 4 different categories of the percentage of fixed elements are identified according to the area of fixed elements of the building plant in relation to the total area of building plant.

2. Placement of Drain Pipes: Drain pipes are located in service shafts or in sleeves around the structural pillars instead of being placed in dedicated drainage ducts.

3. Adjustability of Service Shafts: Adjusting service shafts areas according to alterations in technical and medical requirements. This can be applied through modular construction

4. Grouped Vertical Circulation Elements: Clustering the vertical core circulation elements together, so that the rest of the floor space is contiguous and open. Grouping vertical secondary system networks (such as electrical risers & low current) with the circulation cores is advisable.

Note: There are various types of service shafts: mechanical, electrical and other medical special ones such as (firefighting, drainage/plumbing, HVAC, power, low current, medical gases, linen chute, garbage chute, PTS chute "horizontal and vertical", natural gas)

Assessment Tool Scales – Technology

Evaluation Parameters	Analysis Parameters	Score	Assigned Score
Technology	Dry Assembly Technique	4	
	Mixed Assembly Technique	2	
	Wet Assembly Technique	0	
	Internal Partitions: Modular Panels	+1	
	Internal Partitions: Panels Set Up with Technical Installations	+1	
	Internal Partitions: Movable/Retractable	+1	
	Internal Partitions: Framed Construction	+1	
	Internal Partitions: Prefabricated Panels*	+2*	
	Internal Partitions: Dry Walls Built In Situ*	+1*	
Technology Total Assigned Score			/10

Criteria

1. Assembly Techniques: The 3 different categories of Assembly are identified according to the used technique. In the Dry Assembly, internal dry partition walls are created by modules that are assembled with mechanical fixing technologies that do not require joining materials as glues. While the Mixed Assembly is an integration between the 2 technique.

2. Internal Partitions: Modular Panels: Technique that allows rapid and relatively simple installation, while providing flexibility for easy adaptation to any constructions type. In some embodiments, the modular panel assembly involves panels which are prefabricated with panel connectors.

3. Internal Partitions: Panels Set Up with Technical Installations: Internal non load-bearing partitions, created by separate prefabricated, self-supporting elements installed with channels that accommodate technical network.

4. Internal Partitions: Movable/Retractable: Internal movable walls can be based on sliding, accordion or telescopic partitions. They allow various flexible ways for the use of space by joining/ separating different adjacent rooms.

5. Internal Partitions: Framed Construction: Assembling Internal Partitions in a framed modular structure that allows partition walls to be altered easily.

6. Internal Partitions: Prefabricated Panels: Internal Partitions with connections that are easy to mount and take down, in which the are minimum technical installations and standardized connections. This type require only assembly work.

7. Internal Partitions: Dry Walls Built In Situ: Similar to Prefabricated Panel, yet it requires construction work that takes place on site which cause disturbance to the facility users.

Assessment Tool Scales – Exchange of Large Equipments

Evaluation Parameters	Analysis Parameters	Score	Assigned Score
Exchange of Large Equipments	Only Needs Disassembly of Facade Panels	8	
	Disassembly of Facade Panels and of Internal Partitions	4	
	Partial demolitions	0	
	Large Equip.: In Ground Floor/ Floor with Direct Contact to Outside	+1	
	Equipments Spaces with Redundancy	+1	
Exchange of Large Equipments Total Assigned Score			/10

Criteria

1. Complexity of Exchangeability: The 3 different categories of the Complexity of Exchangeability of large equipments are identified according to the nature of intervention carried out to the building.

2. Large Equip.: In Ground Floor/ Floor with Direct Contact to Outside: Locating large equipments either in the ground floor or in a floor that can be directly accessed from the outside in order to simplify the exchangeability process.

3. Equipments Spaces with Redundancy: Equipments rooms to be designed with extra spaces to be adaptable to accommodate advancements of medical equipments, as it is impossible to foresee the physical space required for the latest equipments

Assessment Tool Scales – Functionality

Evaluation Parameters	Analysis Parameters	Score	Assigned Score
Functionality	Generic/Universal Rooms	+4	
	Space Standardization	+2	
	Double Function	+1	
	Overflow Design	+1	
	Loose Fit	+1	
	Furniture/Equipment Flexibility	+1	
Functionality Total Assigned Score			/10

Criteria

1. Generic/Universal Rooms: Prototype spaces with a modular grid that provides a consistent approach that is efficient in cost, operation, staffing and build-ability limiting the number of specialized spaces. This will permit a change of use between departments if the need arises.

2. Space Standardization: Measured spaces that many features are individually identified. Characteristics of a standardized spaces as: standardized rooms dimensions, modular units, identical room patterns, modular detailing and standard components designed to perform multiple functions based on the need.

3. Double Function: This concept refers to plans which allow for changes in operating mode as a function of management rather than physical building change. It represents a change to the size without any need to expand the unit or make any physical changes.

4.Overflow Design: Functions that are designed to serve as overflow for other functions that are subject to fluctuating demand. For instance, Day and ambulatory care areas can be adapted for overnight use in emergencies such as those relating to natural catastrophes.

5. Loose Fit: Planning models which can not only adequately respond to today' s operational policy but have the inherent flexibility to adapt to a range of alternative. Simple, well proportioned, regular shaped rooms with good access to simple circulation networks that are uncomplicated by a desire to create interest, should be considered..

6. Furniture/Equipment Flexibility: Using furniture that can be adjusted and moved to enable personalization and modification in the room. And/or using standardized equipment to permit movement into different areas for flexibility of function. Using portable equipment where possible, when equipment must be fixed, design for other functions in room to maximize use.
